# Electroacupuncture Acutely Improves Cerebral Blood Flow and Attenuates Moderate Ischemic Injury via an Endothelial Mechanism in Mice

**DOI:** 10.1371/journal.pone.0056736

**Published:** 2013-02-13

**Authors:** Ji Hyun Kim, Kyung Ha Choi, Young Jung Jang, Sun Sik Bae, Byung-Cheul Shin, Byung Tae Choi, Hwa Kyoung Shin

**Affiliations:** 1 Division of Meridian and Structural Medicine, School of Korean Medicine, Pusan National University, Yangsan, Gyeongnam, Republic of Korea; 2 Department of Pharmacology, School of Medicine, Pusan National University, Yangsan, Gyeongnam, Republic of Korea; 3 Division of Clinical Medicine, School of Korean Medicine, Pusan National University, Yangsan, Gyeongnam, Republic of Korea; University of Münster, Germany

## Abstract

Electroacupuncture (EA) is a novel therapy based on traditional acupuncture combined with modern eletrotherapy that is currently being investigated as a treatment for acute ischemic stroke. Here, we studied whether acute EA stimulation improves tissue and functional outcome following experimentally induced cerebral ischemia in mice. We hypothesized that endothelial nitric oxide synthase (eNOS)-mediated perfusion augmentation was related to the beneficial effects of EA by interventions in acute ischemic injury. EA stimulation at Baihui (GV20) and Dazhui (GV14) increased cerebral perfusion in the cerebral cortex, which was suppressed in eNOS KO, but there was no mean arterial blood pressure (MABP) response. The increased perfusion elicited by EA were completely abolished by a muscarinic acetylcholine receptor (mAChR) blocker (atropine), but not a β-adrenergic receptor blocker (propranolol), an α-adrenergic receptor blocker (phentolamine), or a nicotinic acetylcholine receptor (nAChR) blocker (mecamylamine). In addition, EA increased acetylcholine (ACh) release and mAChR M3 expression in the cerebral cortex. Acute EA stimulation after occlusion significantly reduced infarct volume by 34.5% when compared to a control group of mice at 24 h after 60 min-middle cerebral artery occlusion (MCAO) (moderate ischemic injury), but not 90-min MCAO (severe ischemic injury). Furthermore, the impact of EA on moderate ischemic injury was totally abolished in eNOS KO. Consistent with a smaller infarct size, acute EA stimulation led to prominent improvement of neurological function and vestibule-motor function. Our results suggest that acute EA stimulation after moderate focal cerebral ischemia, but not severe ischemia improves tissue and functional recovery and ACh/eNOS-mediated perfusion augmentation might be related to these beneficial effects of EA by interventions in acute ischemic injury.

## Introduction

Despite decades of intense research, current treatments for acute stroke are far from optimal. Electroacupuncture (EA) is a traditional therapy that has been widely applied for treatment of ischemic stroke because it has been shown to improve its outcome in experimental animals [1]–[9] and clinical practice [10]–[13]. Since EA is economical, easily performed, and has few negative side effects, it is clinically applicable for prevention or rehabilitation, but not for acute treatment of stroke. Nevertheless, several studies have shown that acute EA has therapeutic benefits for stroke patients and animal stroke models [4]–[6], [10], but the underlying mechanism is not fully understood and more evidence is needed for acute treatment with EA to be accepted clinically.

Many other factors could have influenced the results of EA studies, including type of stroke, its severity, interval after stroke before treatment began, and amount of time until post treatment assessment [14]. In mild stroke, the differences between the control and the EA groups may be too subtle to detect, even though the EA-treated group may improve more rapidly. Conversely, in severe cases, EA may be minimally effective particularly when the damage to the brain is extensive. Moderate stroke groups probably will show the greatest responsiveness to EA [14], [15]. Although the severity of stroke might be a major confounding factor, few animal studies have been conducted to investigate the effect of EA according to the severity of cerebral ischemia. In this study, to assess the protective effects of EA against moderate and severe ischemic injury, the mice received 20 min-EA stimulation immediately after 60 min- and 90 min-middle cerebral artery occlusion (MCAO).

After cerebral ischemia, restoring blood supply to the ischemic region as rapidly as possible to save the dying neurons, neuroglial cells and vascular endothelial cells is essential for treatment of ischemic cerebrovascular disease. In the past, studies focused on neurons and paid less attention to vascular endothelial cells. After focal cerebral ischemia, dilating the blood vessels, anti-coagulant therapy and thrombolytic therapy obviously improve perfusion of the surrounding tissues, and can promote recovery of the nervous functions. EA has been shown to increase cerebral blood flow (CBF) and improve microcirculation [16]; therefore, improvement of cerebral circulation is a likely mechanism of the beneficial effects of EA in the treatment of cerebral ischemia [17], [18].

It is well-known that nitric oxide (NO) synthesized by endothelial NO synthase (eNOS) plays a pivotal role in maintaining CBF in the ischemic cortex. Under most conditions, stimulation of eNOS activity is protective [19], whereas its inhibition or genetic deletion is detrimental in animal models of stroke [20]. Accordingly, it is possible that EA augments eNOS activity in the cortex, thereby increasing cerebral perfusion. We addressed these questions by exploring the mechanism of perfusion improvement in the cerebral cortex by testing EA stimulation in eNOS knock out (KO) mice.

In this study, we investigated whether acute EA stimulation simultaneously targeting Baihui (GV20) and Dazhui (GV14), improved tissue and functional outcome following experimentally induced cerebral ischemia in mice and, if so, what mechanism mediates this protective effect. We hypothesized that eNOS-mediated perfusion augmentation might be related to the beneficial effects of EA via interventions in acute ischemic injury.

## Materials and Methods

### General surgical preparation

Male mice (C57BL/6J and eNOS deficient, 20–25 g) were housed under diurnal lighting conditions and allowed food and tap water *ad libitum*. This study was carried out in strict accordance with the recommendations in the Guide for the Care and Use of Laboratory Animals of the National Institutes of Health. The protocol was approved by the Pusan National University Institutional Animal Care and Use Committee (Permit Number: PNU-2011-000420). Computer-generated randomization was conducted by SigmaPlot 11.2 (Systat Software Inc, San Jose, CA) for allocating to EA group or control group. After getting the random number by computer-generated randomization, C57/BL6J male mice were allocated either EA group or control group in a blinded fashion. Anesthesia was achieved by face mask-delivered isoflurane (2% induction and 1.5% maintenance, in 80% N_2_O and 20% O_2_). The femoral artery was catheterized for measurement of the mean arterial blood pressure (MABP) using an MLT844 physiological pressure transducer (AD Instruments, Medford, MA). The data were continuously recorded using a PowerLab data acquisition and analysis system (AD Instruments, Medford, MA) and stored in a computer. The depth of anesthesia was checked by the absence of cardiovascular changes in response to a tail pinch. Rectal temperature was kept at 36.5°C–37.5°C using a Panlab thermostatically controlled heating mat (Harvard Apparatus, Holliston, MA). Arterial blood gases and pH were measured before and after EA using i-Stat System (Abbott, Abott Park, IL). Relative CBF was measured in C57BL/6J and eNOS KO using a PeriFlux Laser Doppler System 5000 (Perimed, Stockholm, Sweden). A flexible 0.5-mm fiberoptic probe was affixed to the skull over the brain cortex supplied by the middle cerebral artery (MCA) (2 mm posterior and 7 mm lateral to bregma). CBF measurement was conducted for 5 min before EA stimulation, 20 min during EA and 20 min after EA, giving a total of 45 min. Baseline CBF values measured before EA were defined as 100% flow.

### Electroacupuncture stimulation

**Figure 1:**
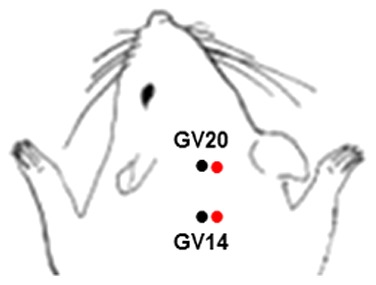
Mouse schematic showing the location of the acupuncture points used in the study. GV20 stands for ‘Baihui’, which is located at the right midpoint of the parietal bone, GV14 stands for ‘Dazhui’, which is located on the posterior midline and in the depression below the spinous process of the seventh cervical vertebra. The control groups received the same electrical stimulation at a lateral point (red) to aforementioned acupoint (black) by approximately 1 mm apart.

Animals were anesthetized with isoflurane to avoid restraint stress. The transpositional method was used to determine the acupoints in mice. In this method, the veterinary acupoints are located by transforming human acupoints onto the animal anatomy [21]. The acupoint Baihui (GV20), which is located at the right midpoint of the parietal bone, and Dazhui (GV14), which is located on the posterior midline and in the depression below the spinous process of the seventh cervical vertebra, were stimulated as standard criteria in mouse (Figure 1). Acupuncture needles (0.18×30 mm) were inserted into GV20 and GV14, after which the acupoints were stimulated at an intensity of 1 mA and a frequency of 2 Hz for 20 min using a Grass S88 electrostimulator (Grass Instrument Co., West Warwick, RI). The current delivered was monitored at all times using Digital Storage Oscilloscope (Tektronix Inc., Beaverton, OR). The intensity was maintained just below the level that induced visible muscle contraction. The control groups received the same electrical stimulation at a lateral point to aforementioned acupoint (Figure 1). To assess the protective effects of EA against ischemic injury, the mice received 20 min-EA stimulation immediately after the onset of occlusion.

### Measurement of acetylcholine in the cerebral cortex

Normal mice were deeply anesthetized with thiopental sodium at 20 min after the end of EA treatment, after which the cortical brain tissues were collected. The amount of acetylcholine (ACh) from brain tissues was then determined using a commercially available ELISA kit according to the manufacturer's instructions (Abcam, UK, Cambridge, UK).

### Immunohistochemistry

Twenty min after the end of EA treatment, mice were deeply anesthetized with thiopental sodium and subsequently perfused transcardially with cold PBS followed by 4% paraformaldehyde for fixation. The brain of each mouse was then removed and further fixed for 24 h in 4% paraformaldehyde at 4°C followed by cryoprotection in 20% sucrose for 72 h at 4°C. The isolated brains were frozen in an optical cutting temperature medium for frozen tissue specimens (Sakura Finetek, Torrance, CA) and then stored in the freezer at −80°C until examined. The frozen brains were cut at a thickness of 14 µm using a Leica CM 3050 cryostat (Leica Microsystes, Wetzlar, Germany). For immunohistochemistry, endogenous peroxidase activity was quenched by incubation with 0.3% H_2_O_2_ and non-specific antibody binding was blocked with CAS Block (Zymed Laboratories, Inc., San Francisco, CA) for 10 min. The preparations were then incubated overnight at 4°C with antibody against muscarinic ACh receptor (mAChR) M3 (Santa Cruz Biotechnology, Santa Cruz, CA), after which they were incubated with an avidin-biotin peroxidase complex solution (Vectastain Elite ABC kit, Vector Laboratories, Burlingame, CA) for 1 h. The immunoreaction products were visualized with a solution of 3,3′-diaminobenzidine tetrahydrochloride (diaminobenzidine substrate kit, Vector laboratories), then counterstained with hematoxylin (Sigma-Aldrich, St. Louis, MO). All samples were visualized using a light microscope (Carl Zeiss, Jena, Germany).

### Focal cerebral ischemia

Focal cerebral ischemia was induced by occluding the MCA using a previously described intraluminal filament technique [22]. Briefly, MCA occlusion was induced by a silicon-coated 7-0 monofilament in the internal carotid artery and the monofilament was advanced to occlude the MCA. In all animals, regional CBF was measured using a PeriFlux Laser Doppler System 5000 (Perimed) with a flexible probe to confirm the achievement of consistent and similar levels of ischemic induction. The filament was withdrawn 60 min and 90 min after occlusion and reperfusion was confirmed using a laser Doppler. The surgical wound was sutured and mice were allowed to recover from anesthesia. The brains were then removed at 24 h after MCA occlusion. Cerebral infarct size was determined on 2,3,5-triphenyltetrazolium chloride (TTC)-stained, 2-mm-thick brain sections. Infarction areas were quantified using the iSolution full image analysis software (Image & Microscope Technology, Vancouver, Canada). To account for and eliminate the effects of swelling/edema, infarction volume was calculated via an indirect measurement by summing the volumes of each section according to the following formula: contralateral hemisphere (mm^3^) - undamaged ipsilateral hemisphere (mm^3^).

### Neurological score

Neurological deficit was scored in each mouse at 24 h after the ischemic insult in a blinded fashion according to the following graded scoring system: 0 = no deficit; 1 = forelimb weakness and torso turning to the ipsilateral side when held by tail; 2 = circling to the affected side; 3 = unable to bear weight on the affected side; and 4 = no spontaneous locomotor activity or barrel rolling [23].

### Evaluation of motor function

Vestibulo-motor function was assessed using a wire-grip test 24 h after cerebral ischemia [24]. Briefly, mice were placed on a metal wire (45 cm long) suspended 45 cm above protective padding and allowed to traverse the wire for 60 sec. The latency for which a mouse remained on the wire within a 60-sec interval was measured, and the wire grip score was quantified using the following 5-point scale: being unable to remain on the wire for less than 30 sec = 0; failure to hold on to the wire with both sets of fore paws and hind paws together = 1; holding on to the wire with both forepaws and hind paws but not the tail = 2; holding on to the wire using the tail along with both forepaws and both hind paws = 3; moving along the wire on all four paws plus tail = 4; a score of 4 points and also ambulating down one of the posts used to support the wire = 5. Tests were performed in triplicate and the average value was calculated for each mouse on each test day.

### Data analysis

First we checked the data whether normally distributed by conducting normality test using SigmaPlot 11.2 (Systat Software Inc). When data were normally distributed, we planned to use parametric analysis method and if not, we planned to conduct nonparametric statistical analysis. All the data showed normal distribution, therefore we conducted parametric analysis. The data are expressed as mean ± standard error of mean (SEM). Two-way analysis of variance (ANOVA) for repeated measures followed by Student-Newman-Keuls multiple comparisons test were used to compare CBF between the control and EA and between the vehicle and drug-treated groups. Unpaired t-test was used to compare acetylcholine, infarct volume, neurological score and wire grip test between control and EA group. *P*<0.05 was considered statistically significant. Statistical analysis was performed using SigmaPlot 11.2 (Systat Software Inc).

## Results

### Physiological parameters

There were no significant differences in physiological parameters during EA treatment (at the onset of EA and the end of EA). Arterial blood gases (pO_2_, pCO_2_ and pH) and MABP remained in the normal range throughout the experimental period (Table 1).

**Table 1:** Physiological parameters.

	Control (N = 10)		EA group (N = 10)		eNOS KO (N = 10)	
	Before EA	After EA	Before EA	After EA	Before EA	After EA
MABP	81.69±1.25	80.05±2.52	82.96±2.00	80.40±1.41	98.63±1.90	94.70±2.10
pH	7.35±0.05	7.31±0.01	7.34±0.01	7.28±0.01	7.31±0.01	7.29±0.02
pO_2_	117.33±6.89	100.00±2.31	117.33±7.86	114.00±6.00	119.11±4.46	108.38±6.95
pCO_2_	37.37±5.28	41.27±1.01	41.77±3.03	42.40±1.10	37.22±1.16	41.98±2.11

Values are the means ± SEM. MABP (mean arterial blood pressure), pO_2_, and pCO_2_ are expressed in mmHg.

### Cerebral perfusion and MABP responses to EA stimulation in the cerebral cortex

**Figure 2:**
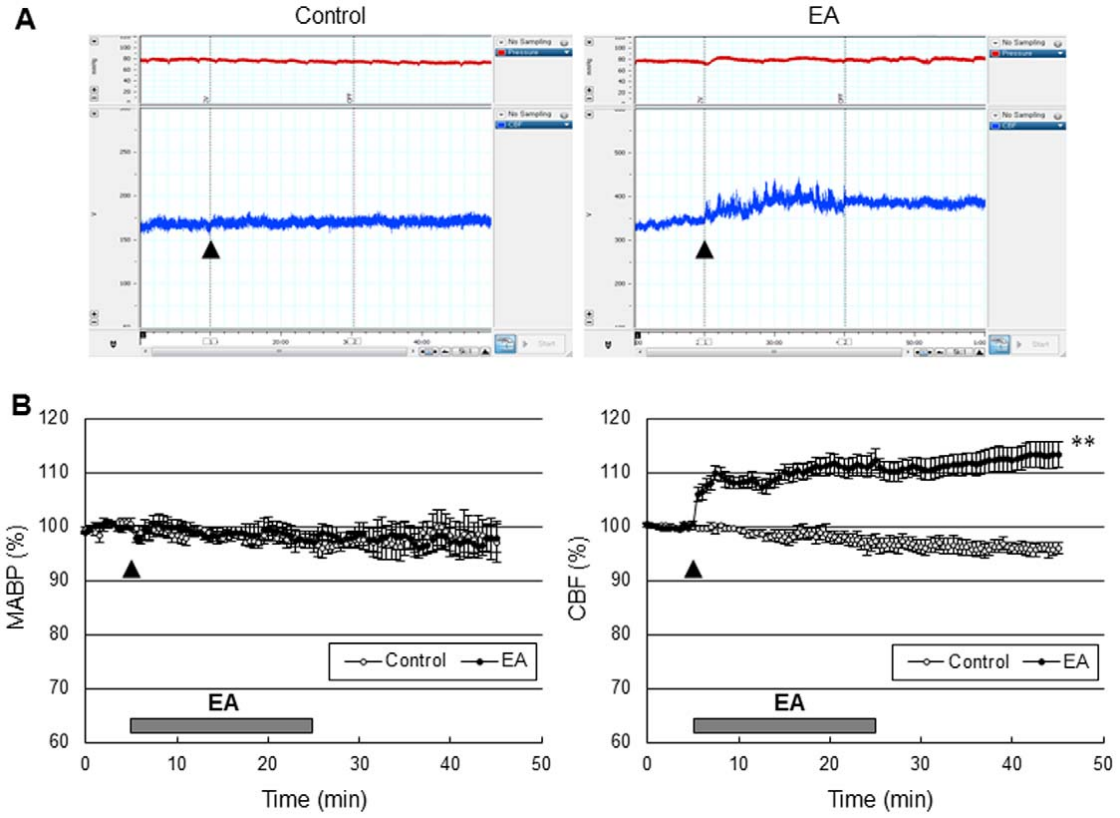
EA at Baihui (GV20) and Dazhui (GV14) increased cerebral perfusion in the cerebral cortex, not blood pressure. (A) Mean arterial blood pressure (MABP, red) and cerebral blood flow (CBF, blue) time course after EA (arrow) from a control group and EA group, and (B) the average of ten experiments. The control groups received the same electrical stimulation at non-acupuncture points. The horizontal bar represents the EA stimulation period. MABP and CBF measurement were conducted for 5 min before EA stimulation, 20 min during EA and 20 min after EA, lasting a total of 45 min. EA significantly increased cerebral perfusion (**, *P*<0.01 vs. control group, two-way ANOVA for repeated measures). Vertical error bars indicate ± SEM.

EA stimulation of GV20 and GV14 produced an increase in perfusion in the cerebral cortex, but there was no MABP response (Figure 2). Perfusion started to increase 10 sec after the onset of EA stimulation, gradually increased to 12.4±1.9% of the baseline during EA stimulation and remained at the increased levels for about 20 min after the end of EA stimulation. The control group did not produce perfusion or MABP responses.

### Effects of adrenergic or cholinergic receptor blockers on EA-induced perfusion changes

**Figure 3:**
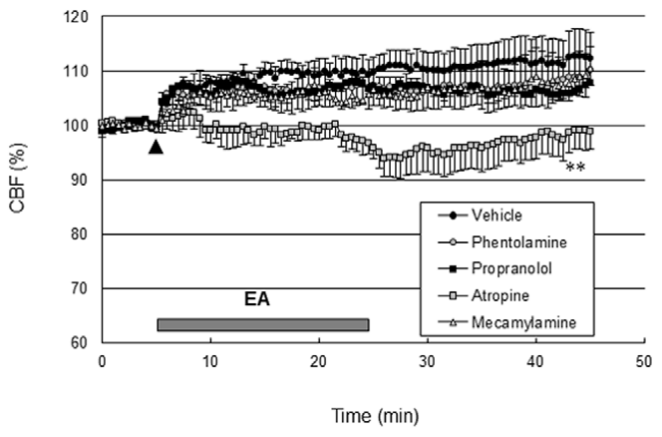
EA increased cerebral perfusion via the muscarinic acetylcholine receptor. Propranolol (2 mg/kg), a β-adrenergic receptor blocker, phentolamine (10 mg/kg), an α-adrenergic receptor blocker, mecamylamine (2 mg/kg), a blood brain barrier permeable nicotinic acetylcholine receptor blocker, or atropine (5 mg/kg), a blood brain barrier permeable muscarinic acetylcholine receptor blocker were administered intravenously 30 min prior to EA stimulation (arrow). The vehicle groups received saline intravenously in the same volume as the blockers. The horizontal bar represents the EA stimulation period. CBF measurement was conducted for 5 min before EA stimulation, 20 min during EA and 20 min after EA, lasting a total of 45 min. The perfusion responses elicited by EA were almost abolished by atropine (**, *P*<0.01 vs. vehicle group, two-way ANOVA for repeated measures, N = 4) not propranolol, phentolamine or mecamylamine. Vertical error bars indicate ± SEM.

The basal perfusion level was not significantly changed following the administration of adrenergic and cholinergic receptor blockers. As shown in Figure 3, the perfusion response elicited by EA did not change following intravenous injection of either propranolol (2 mg/kg, Sigma-Aldrich), a β-adrenergic receptor blocker, phentolamine (10 mg/kg, Sigma-Aldrich), an α-adrenergic receptor blocker, or mecamylamine (2 mg/kg, Sigma-Aldrich), a blood brain barrier permeable nicotinic AChR (nAChR) blocker. However, EA-induced perfusion increase was totally abolished after intravenous injection of atropine (5 mg/kg, Sigma-Aldrich), a blood brain barrier permeable mAChR blocker (*P*<0.01 vs. vehicle group, two-way ANOVA for repeated measures; Figure 3). These data suggest that EA increased perfusion via a mAChR.

### Acetylcholine production and muscarinic receptor expression in responses to EA stimulation in the cerebral cortex

**Figure 4:**
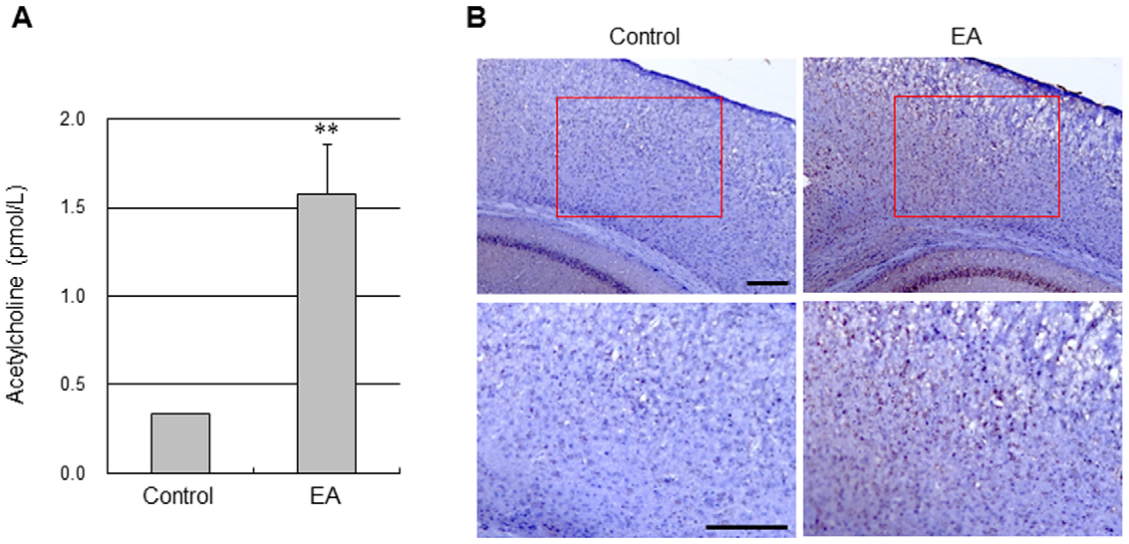
EA increased acetylcholine production and muscarinic acetylcholine receptor expression in the cerebral cortex. (A) Acetylcholine levels in the cerebral cortex 20 min after the end of EA stimulation were analyzed by ELISA. EA significantly increased acetylcholine (**, *P*<0.01 vs. control group, unpaired t-test, N = 4). Vertical error bars indicate ± SEM. (B) Representative immunohistochemical staining photographs showed muscarinic acetylcholine receptor (mAChR) M3-positive cell expression 20 min after the end of EA stimulation in the cerebral cortex of mice. The red rectangle represents the imaging field. EA stimulation increased mAChR M3 expression in the cerebral cortex. Scale bar = 100 µm.

EA stimulation of GV20 and GV14 significantly increased the cortical ACh levels (1.57±0.28 vs. 0.33±0.00 pmol/L, EA group and control group; *P*<0.01, unpaired t-test; Figure 4A). Furthermore, a large number of mAchR M3-positive cells in the cerebral cortex were seen in the EA group, whereas only a small amount of mAChR M3-positive cells were observed in the control group (Figure 4B).

### Cerebral perfusion responses to EA stimulation in eNOS KO

**Figure 5:**
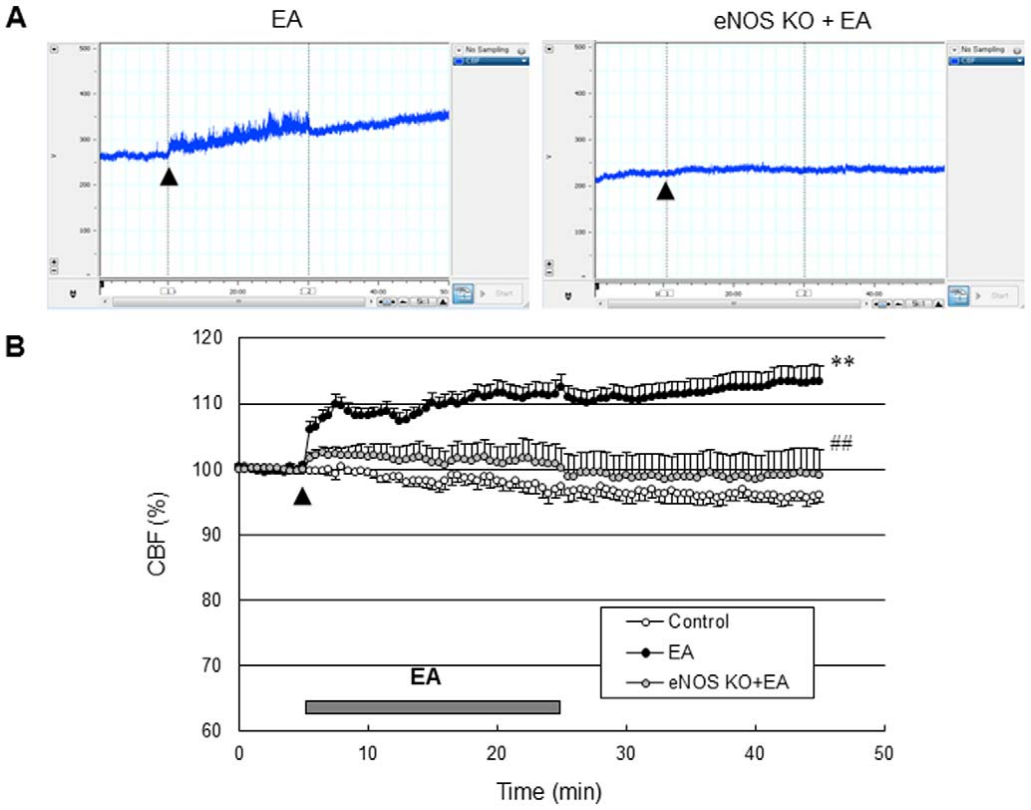
Cerebral perfusion responses elicited by EA abolished in eNOS KO. (A) Cerebral blood flow (CBF) time course after EA (arrow) from a representative experiment from the EA group of C57BL/6J and from the EA group of eNOS KO, and (B) the average of ten experiments. The control groups received the same electrical stimulation at non-acupuncture points. The horizontal bar represents the EA stimulation period. CBF measurement was conducted for 5 min before EA stimulation, 20 min during EA and 20 min after EA, lasting a total of 45 min. The cerebral perfusion response elicited by EA was significantly attenuated in eNOS KO (**, *P*<0.01 vs. control group; ^##^, *P*<0.01 vs. EA group of C57BL/6J, two-way ANOVA for repeated measures). Vertical error bars indicate ± SEM.

To examine the contribution of eNOS signaling to the EA-induced perfusion responses, the effect of EA on perfusion was tested in eNOS KO mice. We found that the perfusion response elicited by EA stimulation in eNOS KO was significantly attenuated (*P*<0.01 vs. EA group of C57BL/6J, two-way ANOVA for repeated measures; Figure 5), suggesting that the cerebral hemodynamic effects of EA are dependent on eNOS.

### Effect of acute EA treatment on tissue outcome and functional outcome in focal cerebral ischemia

**Figure 6:**
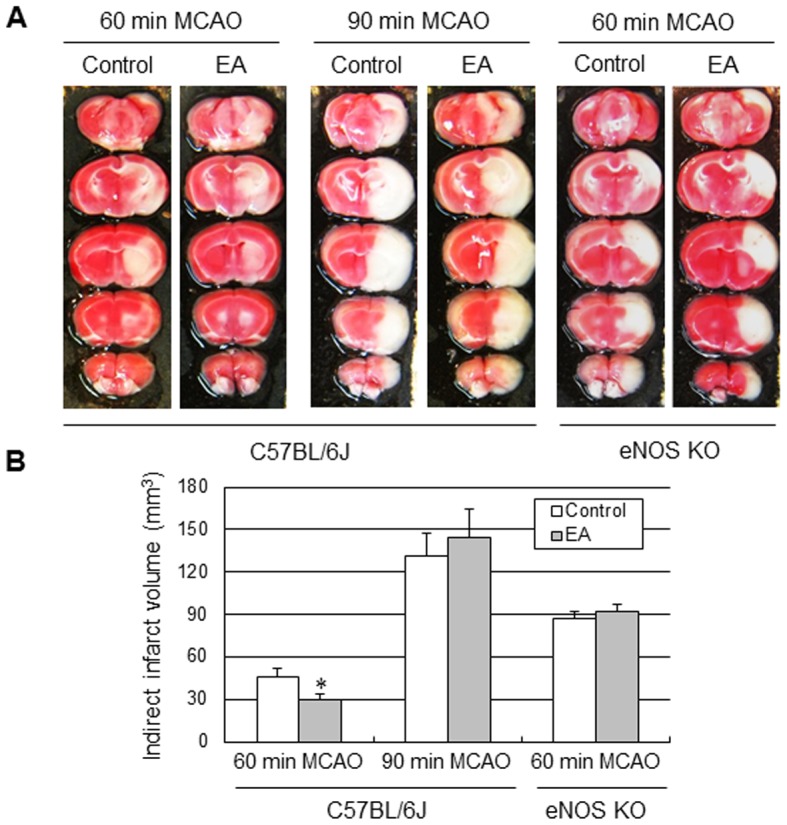
EA improved tissue outcome in moderate ischemic injury, but not severe ischemic injury. Mice were subjected to 60 min and 90 min MCA occlusion followed by 24-h reperfusion. The mice received 20 min-EA stimulation immediately after the onset of occlusion. (A) Representative photographs of coronal brain sections following infarction stained with 2,3,5-triphenyltetrazolium chloride. The red area is healthy tissue and the white area is infarct tissue. (B) Quantification of indirect infarct volume at 24 h after ischemia. Infarct volume was calculated by integrating the infarct area in 2-mm-thick coronal slices. Results are expressed as means ± SEM. *, *P*<0.05 vs. control group, unpaired t-test, N = 6.

**Figure 7:**
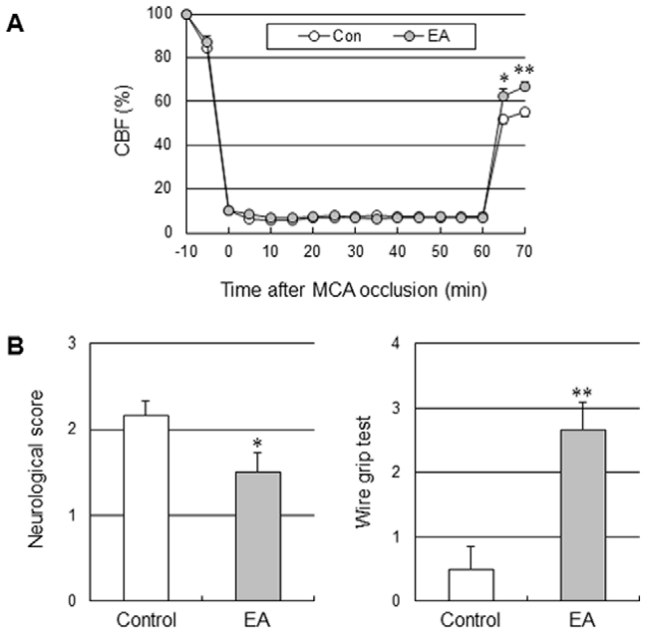
EA improved cerebral perfusion, the neurological function and motor function in moderate ischemic injury. (A) Effect of EA on perfusion measured by laser Doppler flowmetry in moderate ischemic injury (B) Neurological deficit and motor deficit were assessed 24 h after ischemia. Neurological function was assessed by neurological score, and vestibule-motor function was assessed by the wire grip test. Acute EA treatment after ischemia significantly improved the neurological function and motor function. The results are expressed as means ± SEM. *, *P*<0.05 and **, *P*<0.01 vs. control group unpaired t-test, N = 6.

To determine whether the acute increase in perfusion in response to EA improved tissue and functional outcome in cerebral ischemic injury, the mice received EA stimulation immediately after onset of the ischemic event. The control groups received the same electrical stimulation at a lateral point to aforementioned acupoint. Two out of 8 mice in the control group died (25%), and 1 out of 7 mice (14%) in the EA group died at 24 h after 90 min-occlusion, and there were no losses of mice in other surgical groups. Acute EA stimulation significantly reduced infarct volume by 34.5% when compared to the control group mice at 24 h after 60 min-MCA occlusion (moderate ischemic injury), but not after 90-min MCA occlusion (severe ischemic injury), suggesting that EA might be effective for treatment of moderate ischemic injury, but not severe ischemic injury (Figure 6). In addition, the impact of EA on moderate ischemic injury was totally abolished in eNOS KO, suggesting that the cerebroprotective effects of acute EA are dependent on eNOS. EA stimulation did not change cortical perfusion during filament-induced MCAO, but significantly increased perfusion during 10 min of reperfusion (Figure 7A). Consistent with a smaller infarct size, acute EA stimulation showed prominent improvement of neurological function and vestibule-motor function (Figure 7B).

## Discussion

EA is a potential therapeutic strategy to improve functional outcome in acute ischemic stroke. Here, we provide evidence that acute EA stimulation at Baihui (GV20) and Dazhui (GV14) after moderate focal cerebral ischemia improves tissue and functional recovery, and that ACh/eNOS-mediated perfusion augmentation might be related to these beneficial effects of EA in response to interventions in moderate ischemic injury. Our results showed that EA stimulation increased perfusion in the cerebral cortex, which was almost abolished by a mAChR blocker. In addition, EA increased acetylcholine release and mAChR M3 expression in the cerebral cortex. Acute EA stimulation after occlusion significantly reduced moderate ischemic injury, which was suppressed in eNOS KO, suggesting that the cerebrovascular protective effects of EA are dependent on eNOS. Consistent with a smaller infarct size, acute EA treatment showed prominent improvement of neurological function and vestibule-motor function. These findings support the view that acute EA treatment improves cerebrovascular damage in the acute stage of moderate focal cerebral ischemia, at least partially because of an increase of cerebral perfusion in an ACh/eNOS-dependent fashion.

EA is a novel therapy based on traditional acupuncture combined with modern electrotherapy that exets many effects including analgesia, promotion of homeostasis, and changes in the microcirculatory network [25], as well as improvements in brain circulation [16]. EA has been widely applied for treatment of ischemic stroke as it has been shown to improve the outcome of ischemic stroke in experimental [1]–[9] and clinical practice [10]–[13]. However, most EA studies were performed to investigate the preventive and chronic effects of EA on ischemic cerebral disease. In recent years, several studies have shown that EA also has therapeutic benefits of acute treatment in focal cerebral ischemia [4]–[6]. Based on these findings, we investigated whether acute EA stimulation improves tissue outcome and functional outcome following experimentally induced cerebral ischemia in mice. We delivered EA stimulation to GV20 and GV14 immediately after ischemia onset to maximize its potential benefit. Acute EA stimulation after occlusion significantly reduced infarct volume by 34.5% when compared to the control group mice at 24 h after 60 min-MCA occlusion (moderate ischemic injury), but not after 90-min MCA occlusion (severe ischemic injury). Consistent with a smaller infarct size, acute EA stimulation led to great improvement of neurological function and vestibule-motor function. These findings suggest that EA has a therapeutic benefit when administered early after stroke onset in moderate ischemic injury; however, more work is needed to determine the therapeutic window for EA to improve tissue outcome and functional outcome.

Although acute EA treatment could induce a protective effect against cerebral ischemic injury, there are several characteristics of EA treatment that differ from other treatment methods, especially the acupoint specificity. The Baihui (GV20) and Dazhui (GV14) acupoints were chosen because the theory of meridians in traditional Korean medicine indicates that they are closely related to the brain and spinal cord and they were commonly used to treat stroke in ancient Korea. Additionally, these acupoints protect against hypoxic-ischemic brain damage in immature rats [26] and facilitate the recovery of post-ischemic behavioral dysfunction [27]. In the present study, acute EA stimulation at GV20 and GV14 after moderate focal cerebral ischemia improves tissue and functional recovery. However, this result raises the concern that the present findings were not mediated by acupuncture, but by electrical stimulation. Therefore, we compared these results with those observed for control mice that received the same electrical stimulation at non-acupuncture points. This comparison suggested that the effects of EA at GV20 and GV14 cannot be attributed to electrical stimulation alone.

Our understanding of the mechanisms by which EA stimulation in the acute stage of focal cerebral ischemia improved tissue and functional outcome has been limited; however, several mechanisms for the protective effects of acute EA after ischemia have been suggested. EA administered 15 min after occlusion of MCA enhanced the expression of insulin-like growth factor-1, which might be an important mechanism of neuroprotective effects of EA against cerebral ischemia in monkey [4]. Moreover, application of EA immediately after ischemia could increase anti-oxidative activities, thereby reducing neurological deficits in ischemic rats [5], [28]. When EA stimulation was administered immediately after reperfusion, EA attenuated neuronal cell death via regulation of different receptors such as NMDA receptor, and downstream intracellular signaling events inducing phosphatidylinositol 3-kinase (PI3K), Akt and caspase-9 [6], [29], [30]. However the precise mechanism through which this occurred is not fully understood and more evidence is needed for acute treatment with EA to be accepted clinically.

Therapies that reinstate CBF to the ischemic territory are effective in acute stroke, suggesting that CBF is a critical determinant of final stroke outcome. Therefore, it is possible that enhancement of CBF might be the underlying mechanism of EA. A large number of animal studies have shown that EA could increase CBF in ischemic injury. The local CBF in the striatum decreased during MCAO, whereas EA improved the CBF significantly in focal cerebral ischemic monkey [31]. Appropriate EA treatment protects the brain from cerebral ischemia by increasing blood flow to the ischemic brain region via rapid regulation [17]. The results of a preliminary human series were promising as indicated by increased CBF in patients with stroke after acupuncture using SPECT scanning [18] and in patients with vascular dementia during acupuncture using fluorodeoxy glucose positron emission tomography (FDG-PET) [32]. We evaluated CBF at the cortex overlying the MCA territory during and after EA stimulation, and showed that perfusion started to increase 10 sec after the onset of EA stimulation, gradually increased during EA stimulation and remained at the increased levels for about 20 min after the end of EA stimulation. However, EA stimulation at GV20 and GV14 did not cause MABP responses, and even improved perfusion, suggesting that MABP was not involved in the increase of cerebral perfusion observed in this study. Our data are consistent with previous reports that EA did not change MABP in control animals, although EA reduced MABP in experimental hypertensive animals [33]. It also has been reported that acupuncture-like stimulation increases cortical blood flow by activating cholinergic vasodilators, and that increase of blood flow is independent of BP [16]. In light of the potential detrimental effect of systemic vasodilation and hyperemia on CBF in acute stroke, EA stimulation at GV20 and GV14 may be more efficacious in stroke therapy.

The mechanism of improved perfusion in the cerebral cortex by EA stimulation is unclear. Several lines of evidence have indicated that the central cholinergic system is sensitive to ischemia, and that even mild hypoxia may impair the synthesis of ACh [34]. Cholinergic receptors are involved in the control of CBF via mediation of vasodilation of cortical microvessels upon stimulation of cholinergic cells in the basal forebrain. One mechanism of EA stimulation is via increasing perfusion by activating the intracranial cholinergic vasodilative system [16]. Consistent with these reports, we found that EA stimulation at GV20 and GV14 significantly increased ACh release in the cerebral cortex (Figure 4A). An endothelium derived relaxing factor acting as a vasodilator during ACh-induced vasodilation of blood vessels has been identified as NO. Several studies employing human and animal models have shown that acupuncture enhances the generation of NO and increases local circulation [35]. We explored the mechanism of CBF improvement in the cerebral cortex by testing EA stimulation at GV20 and GV14 in eNOS KO mice and showed that EA did not augment perfusion, indicating that NO from eNOS is one factor necessary for cholinergic cortical vasodilation induced by EA stimulation. Furthermore, the impact of EA on moderate ischemic injury was totally abolished in eNOS KO, suggesting that the cerebroprotective effects of EA are dependent on eNOS. Therefore, along with previous reports, the results of the present study support the finding that ACh released from the cholinergic nerve following EA stimulation leads to NO release from endothelial cells, and this released NO acts on the smooth muscle of the cerebral blood vessels, resulting in their vasodilation.

The NO-dependent dilation described above could be mediated by either one of two types of ACh receptors identified in brain microvasculature, nicotine [36] and/or muscarinic [37]. Both nAChRs and mAChRs as well as NO have been implicated in these local changes in CBF [38]. However, in some reports, mAChRs, but not nAChRs, mediated a NO-dependent dilation in brain cortical arterioles [39]. Cortical microvessels are endowed with mAChRs [40], and stimulation of those receptors results in microvessel dilation and increased cortical perfusion [41], [42]. Stimulation of mAChRs in cerebrovascular tissue can induce either vasodilation or vasoconstriction depending on the species, the concentration of ACh, the presence of the endothelial cell layer, the subtypes of mAChRs present, and their localization in the vessel wall. For instance, endothelial mAChRs pharmacologically similar to the M3 mAChR subtype seem to mediate dilation of cerebral blood vessels [40], [43], [44]. This endothelial dependent dilation is mediated by NO or an NO derivative [45]. With respect to brain intraparenchymal circulation, ACh has been shown to dilate isolated rat cerebral arterioles [46] as well as microvessels in hippocampal slice preparations [47]. In the present study, the increase in cortical perfusion due to EA stimulation at GV20 and GV14 was significantly attenuated after iv administration of atropine, a mAChR antagonist, suggesting that mAChR contributing to this cortical perfusion response must exist in the cerebral tissues. In contrast, the cortical perfusion response was not affected following iv administration of propranolol, phentolamine or mecamylamine. In addition, EA increased mAChR M3 expression in the cerebral cortex. Taken together, these results suggest that the cholinergic system activated by EA can contribute to the cortical perfusion response via activation of mAChR but not nAChR.

In summary, our data suggest that acute EA stimulation at the GV20 and GV14 acupoints after moderate focal cerebral ischemia has cerebrovascular protective potential mediated, at least in part, via ACh/eNOS-mediated perfusion augmentation. Although further investigation is needed to elucidate stroke subtypes and the therapeutic window within which EA is efficacious, EA may present a novel therapeutic opportunity during acute stroke. At this time, our results provide a scientific basis to better understand the cerebrovascular mechanisms of EA used in acute treatment of cerebral ischemia in the practice of complementary and alternative medicine.

## References

[1] Dong H, Fan YH, Zhang W, Wang Q, Yang QZ, et al (2009) Repeated electroacupuncture preconditioning attenuates matrix metalloproteinase-9 expression and activity after focal cerebral ischemia in rats. Neurol Res 31: 853–858. 19278575 10.1179/174313209X393960

[2] Wang Q, Peng Y, Chen S, Gou X, Hu B, et al (2009) Pretreatment with electroacupuncture induces rapid tolerance to focal cerebral ischemia through regulation of endocannabinoid system. Stroke; a journal of cerebral circulation 40: 2157–2164. 10.1161/STROKEAHA.108.541490 19372445

[3] Wang Q, Li X, Chen Y, Wang F, Yang Q, et al (2011) Activation of epsilon protein kinase C-mediated anti-apoptosis is involved in rapid tolerance induced by electroacupuncture pretreatment through cannabinoid receptor type 1. Stroke; a journal of cerebral circulation 42: 389–396. 10.1161/STROKEAHA.110.597336 21183751

[4] Gao H, Guo J, Zhao P, Cheng J (2006) Influences of electroacupuncture on the expression of insulin-like growth factor-1 following, focal cerebral ischemia in monkeys. Acupunct Electrother Res 31: 259–272. 17608065 10.3727/036012906815844247

[5] Zhong S, Li Z, Huan L, Chen BY (2009) Neurochemical Mechanism of Electroacupuncture: Anti-injury Effect on Cerebral Function after Focal Cerebral Ischemia in Rats. Evid Based Complement Alternat Med 6: 51–56. 10.1093/ecam/nem062 PMC2644276 18955263

[6] Kang KA, Shin ES, Hur J, Hasan MR, Lee H, et al (2010) Acupuncture attenuates neuronal cell death in middle cerebral artery occlusion model of focal ischemia. Neurol Res 32 Suppl 1: 84–87. 20034452 10.1179/016164109X12537002794246

[7] Du Y, Shi L, Li J, Xiong J, Li B, et al (2011) Angiogenesis and improved cerebral blood flow in the ischemic boundary area were detected after electroacupuncture treatment to rats with ischemic stroke. Neurol Res 33: 101–107. 20546685 10.1179/016164110X12714125204317

[8] Ren L, Zhang WA, Fang NY, Wang JX (2008) The influence of electro-acupuncture on neural plasticity in acute cerebral infarction. Neurol Res 30: 985–989. 18671899 10.1179/174313208X325182

[9] Tao J, Xue XH, Chen LD, Yang SL, Jiang M, et al (2010) Electroacupuncture improves neurological deficits and enhances proliferation and differentiation of endogenous nerve stem cells in rats with focal cerebral ischemia. Neurol Res 32: 198–204. 19433012 10.1179/174313209X414506

[10] Si QM, Wu GC, Cao XD (1998) Effects of electroacupuncture on acute cerebral infarction. Acupunct Electrother Res 23: 117–124. 9789586 10.3727/036012998816356562

[11] Liu R (2005) Clinical experience in acupuncture treatment of apoplexy. J Tradit Chin Med 25: 190–192. 16334721

[12] Hopwood V, Lewith GT (2005) Does acupuncture help stroke patients become more independent? J Altern Complement Med 11: 175–177. 15750379 10.1089/acm.2005.11.175

[13] Liu SY, Hsieh CL, Wei TS, Liu PT, Chang YJ, et al (2009) Acupuncture stimulation improves balance function in stroke patients: a single-blinded controlled, randomized study. The American journal of Chinese medicine 37: 483–494. 19606509 10.1142/S0192415X09006990

[14] Shiflett SC (2007) Does acupuncture work for stroke rehabilitation: what do recent clinical trials really show? Top Stroke Rehabil 14: 40–58. 17698457 10.1310/tsr1404-40

[15] Naeser MA, Alexander MP, Stiassny-Eder D, Galler V, Hobbs J, et al (1994) Acupuncture in the treatment of paralysis in chronic and acute stroke patients–improvement correlated with specific CT scan lesion sites. Acupunct Electrother Res 19: 227–249. 7625245 10.3727/036012994816357231

[16] Uchida S, Kagitani F, Suzuki A, Aikawa Y (2000) Effect of acupuncture-like stimulation on cortical cerebral blood flow in anesthetized rats. Jpn J Physiol 50: 495–507. 11120916 10.2170/jjphysiol.50.495

[17] Zhou F, Guo J, Cheng J, Wu G, Xia Y (2011) Electroacupuncture increased cerebral blood flow and reduced ischemic brain injury: dependence on stimulation intensity and frequency. J Appl Physiol 111: 1877–1887. 21836043 10.1152/japplphysiol.00313.2011 PMC3233896

[18] Lee JD, Chon JS, Jeong HK, Kim HJ, Yun M, et al (2003) The cerebrovascular response to traditional acupuncture after stroke. Neuroradiology 45: 780–784. 12942221 10.1007/s00234-003-1080-3

[19] Limbourg FP, Huang Z, Plumier JC, Simoncini T, Fujioka M, et al (2002) Rapid nontranscriptional activation of endothelial nitric oxide synthase mediates increased cerebral blood flow and stroke protection by corticosteroids. J Clin Invest 110: 1729–1738. 12464678 10.1172/JCI15481 PMC151626

[20] Huang Z, Huang PL, Ma J, Meng W, Ayata C, et al (1996) Enlarged infarcts in endothelial nitric oxide synthase knockout mice are attenuated by nitro-L-arginine. J Cereb Blood Flow Metab 16: 981–987. 8784243 10.1097/00004647-199609000-00023

[21] Yin CS, Jeong HS, Park HJ, Baik Y, Yoon MH, et al (2008) A proposed transpositional acupoint system in a mouse and rat model. Res Vet Sci 84: 159–165. 17559895 10.1016/j.rvsc.2007.04.004

[22] Huang Z, Huang PL, Panahian N, Dalkara T, Fishman MC, et al (1994) Effects of cerebral ischemia in mice deficient in neuronal nitric oxide synthase. Science 265: 1883–1885. 7522345 10.1126/science.7522345

[23] Li X, Blizzard KK, Zeng Z, DeVries AC, Hurn PD, et al (2004) Chronic behavioral testing after focal ischemia in the mouse: functional recovery and the effects of gender. Exp Neurol 187: 94–104. 15081592 10.1016/j.expneurol.2004.01.004

[24] Chen T, Liu W, Chao X, Zhang L, Qu Y, et al (2011) Salvianolic acid B attenuates brain damage and inflammation after traumatic brain injury in mice. Brain Res Bull 84: 163–168. 21134421 10.1016/j.brainresbull.2010.11.015

[25] Cummings M (2001) Acupuncture influences cortical blood flow in rats (n = 52). Acupunct Med 19: 64–65. 11471592 10.1136/aim.19.1.64

[26] Liu Y, Zou LP, Du JB, Wong V (2010) Electro-acupuncture protects against hypoxic-ischemic brain-damaged immature rat via hydrogen sulfide as a possible mediator. Neurosci Lett 485: 74–78. 20813156 10.1016/j.neulet.2010.08.068

[27] Han X, Huang X, Wang Y, Chen H (2010) A study of astrocyte activation in the periinfarct region after cerebral ischemia with electroacupuncture. Brain Inj 24: 773–779. 20370384 10.3109/02699051003610482

[28] Siu FK, Lo SC, Leung MC (2004) Electroacupuncture reduces the extent of lipid peroxidation by increasing superoxide dismutase and glutathione peroxidase activities in ischemic-reperfused rat brains. Neurosci Lett 354: 158–162. 14698462 10.1016/j.neulet.2003.10.009

[29] Sun N, Zou X, Shi J, Liu X, Li L, et al (2005) Electroacupuncture regulates NMDA receptor NR1 subunit expression via PI3-K pathway in a rat model of cerebral ischemia-reperfusion. Brain Res 1064: 98–107. 16289403 10.1016/j.brainres.2005.09.060

[30] Wang SJ, Omori N, Li F, Jin G, Zhang WR, et al (2002) Potentiation of Akt and suppression of caspase-9 activations by electroacupuncture after transient middle cerebral artery occlusion in rats. Neurosci Lett 331: 115–118. 12361854 10.1016/s0304-3940(02)00866-2

[31] Gao H, Guo J, Zhao P, Cheng J (2002) The neuroprotective effects of electroacupuncture on focal cerebral ischemia in monkey. Acupunct Electrother Res 27: 45–57. 12044020 10.3727/036012902816026112

[32] Huang Y, Chen J, Htut WM, Lai X, Wik G (2007) Acupuncture increases cerebral glucose metabolism in human vascular dementia. Int J Neurosci 117: 1029–1037. 17613112 10.1080/00207450600936825

[33] Kim DD, Pica AM, Duran RG, Duran WN (2006) Acupuncture reduces experimental renovascular hypertension through mechanisms involving nitric oxide synthases. Microcirculation 13: 577–585. 16990216 10.1080/10739680600885210 PMC1618823

[34] Hartig W, Bauer A, Brauer K, Grosche J, Hortobagyi T, et al (2002) Functional recovery of cholinergic basal forebrain neurons under disease conditions: old problems, new solutions? Rev Neurosci 13: 95–165. 12160262 10.1515/revneuro.2002.13.2.95

[35] Tsuchiya M, Sato EF, Inoue M, Asada A (2007) Acupuncture enhances generation of nitric oxide and increases local circulation. Anesth Analg 104: 301–307. 17242084 10.1213/01.ane.0000230622.16367.fb

[36] Kalaria RN, Homayoun P, Whitehouse PJ (1994) Nicotinic cholinergic receptors associated with mammalian cerebral vessels. J Auton Nerv Syst 49 Suppl: S3–7. 7836682 10.1016/0165-1838(94)90078-7

[37] Estrada C, Hamel E, Krause DN (1983) Biochemical evidence for cholinergic innervation of intracerebral blood vessels. Brain Res 266: 261–270. 6135491 10.1016/0006-8993(83)90657-1

[38] Sato A, Sato Y (1995) Cholinergic neural regulation of regional cerebral blood flow. Alzheimer Dis Assoc Disord 9: 28–38. 7605619 10.1097/00002093-199505000-00007

[39] Elhusseiny A, Hamel E (2000) Muscarinic–but not nicotinic–acetylcholine receptors mediate a nitric oxide-dependent dilation in brain cortical arterioles: a possible role for the M5 receptor subtype. J Cereb Blood Flow Metab 20: 298–305. 10698067 10.1097/00004647-200002000-00011

[40] Dauphin F, MacKenzie ET (1995) Cholinergic and vasoactive intestinal polypeptidergic innervation of the cerebral arteries. Pharmacol Ther 67: 385–417. 8577823 10.1016/0163-7258(95)00022-4

[41] Elhusseiny A, Cohen Z, Olivier A, Stanimirovic DB, Hamel E (1999) Functional acetylcholine muscarinic receptor subtypes in human brain microcirculation: identification and cellular localization. J Cereb Blood Flow Metab 19: 794–802. 10413035 10.1097/00004647-199907000-00010

[42] Hamel E (2004) Cholinergic modulation of the cortical microvascular bed. Prog Brain Res 145: 171–178. 14650915 10.1016/S0079-6123(03)45012-7

[43] Dauphin F, Hamel E (1990) Muscarinic receptor subtype mediating vasodilation feline middle cerebral artery exhibits M3 pharmacology. Eur J Pharmacol 178: 203–213. 2328761 10.1016/0014-2999(90)90476-m

[44] Shimizu T, Rosenblum WI, Nelson GH (1993) M3 and M1 receptors in cerebral arterioles in vivo: evidence for downregulated or ineffective M1 when endothelium is intact. Am J Physiol 264: H665–669. 7681264 10.1152/ajpheart.1993.264.3.H665

[45] Faraci FM, Brian JE Jr (1994) Nitric oxide and the cerebral circulation. Stroke 25: 692–703. 7510430 10.1161/01.str.25.3.692

[46] Dacey RG Jr, Bassett JE (1987) Cholinergic vasodilation of intracerebral arterioles in rats. Am J Physiol 253: H1253–1260. 3479909 10.1152/ajpheart.1987.253.5.H1253

[47] Sagher O, Zhang XQ, Szeto W, Thai QA, Jin Y, et al (1993) Live computerized videomicroscopy of cerebral microvessels in brain slices. J Cereb Blood Flow Metab 13: 676–682. 8314920 10.1038/jcbfm.1993.86

